# Tertiary Lymphoid Organs in Cancer Tissues

**DOI:** 10.3389/fimmu.2016.00244

**Published:** 2016-06-22

**Authors:** Nobuyoshi Hiraoka, Yoshinori Ino, Rie Yamazaki-Itoh

**Affiliations:** ^1^Division of Pathology and Clinical Laboratories, National Cancer Center Hospital, Tokyo, Japan; ^2^Division of Molecular Pathology, National Cancer Center Research Institute, Tokyo, Japan; ^3^Division of Analytical Pathology, National Cancer Center Research Institute, Tokyo, Japan

**Keywords:** tertiary lymphoid organs, cancer, tumor immunology, tissue structure, tumor microenvironment

## Abstract

Tertiary lymphoid organs (TLOs) are induced postnatally in non-lymphoid tissues such as those affected by chronic infections, autoimmune diseases, and chronic allograft rejection, and also in cancer tissues. TLOs are thought to provide important lymphocytic functional environments for both cellular and humoral immunity, similar to lymph nodes or Peyer’s patches. TLOs have a structure similar to that of lymph nodes or Peyer’s patches, including T cell zones, B cell follicles, and high endothelial venules (HEV) without encapsulation. Here, we review recent advances in our knowledge of TLOs in human solid cancers, including their location, structure, methods of evaluation, and clinicopathological impact. We also discuss the formation and/or maintenance of TLOs in cancer tissues in association with the tumor immune microenvironment, cancer invasion, and the tissue structure of the cancer stroma.

## Introduction

Cancer tissue is composed of cancer cells and a stroma (alternatively referred to as the cancer microenvironment or tumor microenvironment), and cancer cells themselves, the cancer stroma, and their interaction can determine the biological behavior of the cancer (1). The cancer stroma is composed of vessels, fibroblasts, immune cells, and an extracellular matrix, making cancer tissue analogous to a form of organ. The host immune system is one of the leading players in the tumor microenvironment (1), and plays a critical role in tumor surveillance (2–4). The host antitumor immune reaction differs according to tumor type, tumor developmental stage, and the tissue from which the tumor develops. For example, colon cancers with many gene mutations such as those with microsatellite instability (MSI) tend to be immunogenic with a higher number of tumor-infiltrating lymphocytes (TILs) (5, 6), and many respond to immunotherapy with immune checkpoint inhibitors, although many patients with colon cancer do not achieve the same degree of effect with any given immunotherapy due to non-immunogenicity of the tumor (7).

Tumor-infiltrating immune cells often represent the host immune reaction. The presence of high numbers of TILs has been found to be a major predictor of favorable clinical outcome in many types of solid cancer, such as colorectal, lung, ovarian, and pancreatic cancers (8, 9). Cells of myeloid lineage, such as macrophages, granulocytes, and mast cells, also infiltrate tumor tissues, especially macrophages, which are usually the most abundant cells infiltrating tumor tissues, and these myeloid cells exert many biological effects in cancer (10–16). These tumor-infiltrating myeloid cells have also been shown to be prognostically significant (9, 12). The tumor immune microenvironment shows a drastic change during the natural history of tumor development and progression, i.e., from an immune reaction to immune tolerance during the progression of multi-step carcinogenesis (17–19). Meanwhile, inflammatory responses affect tumor development at different stages, including initiation, promotion, malignant conversion, invasion, and metastasis (20). Tumor-infiltrating immune cells engage in extensive and dynamic cross-talk with cancer cells, and some of the molecular events that mediate this dialog have been revealed (20). Accumulated evidence suggests that even the same types or subsets of tumor-infiltrating immune cells sometimes have different and opposite effects on patient outcome (8, 21).

The central mechanism involved in cellular immune reactions begins when immature dendritic cells (DCs) take up foreign antigens and then migrate to regional lymph nodes to present the antigens to T cells. The cognate T cells then proliferate and begin to remove the foreign antigens. The B cell-mediated immune response is mainly humoral, and occurs in peripheral lymphoid organs such as lymph nodes or the spleen. Thus, lymph nodes, Peyer’s patches, and the spleen act as a lymphocytic functional environment for both cellular and humoral immunity, and these lymphoid organs are referred to as secondary lymphoid organs (SLOs) or tissues, in contrast to the primary lymphoid organs (PLOs) – the thymus and bone marrow – where lymphocytes are produced and educated. These PLOs and SLOs develop during embryogenesis and early life. Postnatal lymphoid organs with a morphology similar to SLOs are induced to form in non-lymphoid tissues such as those associated with chronic inflammation, chronic allograft rejection, or cancer, and these are known as tertiary lymphoid organs (TLOs) or structures (alternatively, ectopic lymphoid structures). TLOs are generally induced in areas of extensive local activation of cellular and humoral immune responses. TLOs are thought to play roles in immune responses that are similar to those of SLOs (22–26). One type of TLO, bronchus-associated lymphoid tissue (BALT), can independently initiate local B- and T-cell responses (24) and serves as a reservoir of memory B and T cells (23). Mice with BALT are strikingly more resistant to pulmonary infection with a variety of infectious agents than mice without BALT (24, 26). Dieu-Nosjean et al. have observed TLO components in lung cancers that are very similar (but not identical) to those of SLOs, being active in cellular and humoral immune responses (27, 28). In comparison to SLOs, TLOs are located very much closer to, or within, lesions and have similar immune function. Therefore, TLOs act as a front line base or bridgehead on the “immune battlefield.”

## Definition, Location, and Structure of TLOs

Tertiary lymphoid organs can develop in various kinds of inflamed and non-lymphoid tissues including those associated with chronic infections, autoimmune diseases, chronic allograft rejection, and several solid cancers (29–34). TLOs are organized lymphoid structures similar to SLOs, characterized by B-cell follicles, T-cell zones, and specialized vessels known as high endothelial venules (HEVs), although TLOs are not encapsulated and supplied by afferent lymphatics.

Lymphocytic infiltration, lymphocyte trafficking, and lymphocyte homing are accurately regulated by several types of chemotactic factors and adhesion molecules expressed or demonstrated on endothelial cells or along the pathways of lymphocytic movement (35–37). The lymphocyte trafficking system allows appropriate subset of lymphocytes with appropriate activity deploy to appropriate sites, areas, or tissues with appropriate timing. Effector or effector memory lymphocytes infiltrate into inflamed tissues, although large numbers of lymphocytes, particularly naive and central memory lymphocytes, accumulate in TLOs by homing through the HEVs from the blood by a multi-step mechanism that involves l-selectin-, chemokine-, and integrin-mediated lymphocyte-endothelial cell interaction (35, 36, 38). HEVs specifically express l-selectin ligands, including peripheral node addressin (PNAd), which are sulfated sialyl Lewis X molecules whose carbohydrate structures and biological function have been clarified by our group and others (37, 39–44). Chemokines CCL19 and CCL21 are necessary for the recruitment and disposition of T cells and DCs within lymphoid tissue, and chemokine CXCL13 functions in the recruitment and disposition of B cells. These chemokines are also involved in lymphoid neogenesis (29–34).

### Location of TLOs in Cancer Tissues

**Figure 1:**
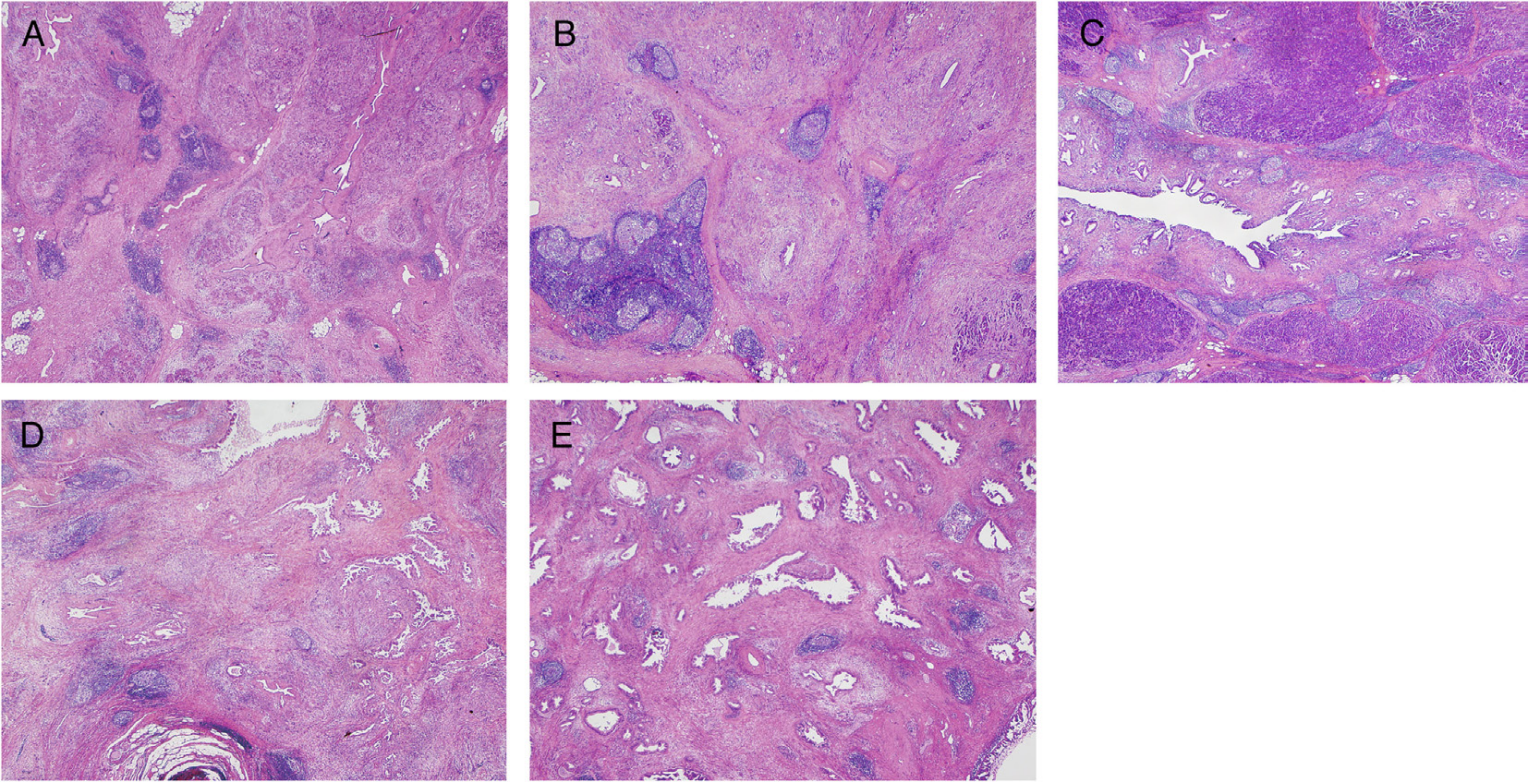
**Histological features of TLOs (45) in chronic pancreatitis (A), IgG4-related lymphoplasmacytic sclerosing pancreatitis (autoimmune pancreatitis) (B,C), and pancreatic ductal adenocarcinoma (PDAC) (D,E)**. TLOs are distributed evenly in inflamed tissues **(A,B)** and sometimes concentrated near the target structure (around ducts) **(C)**. In contrast, cancer tissue is surrounded by peritumoral TLOs **(D)** and a rare pancreatic cancer case has intratumoral TLOs **(D)**. Common PDAC has a paucity of vessels and lacks intratumoral TLOs, although limited cases do have intratumoral TLOs that are richer in tumor-infiltrating lymphocytes and retain relatively intact vascular networks consisting of arterioles, venules, and capillaries without cancer invasion.

Tumor-associated TLOs can be located peritumorally or intratumorally (Figure 1). The majority of TLOs in cancer tissues develop in peritumoral areas, and are characterized as TLOs at the invasive front (or invasive margin), forming a wall around the cancer tissue. Peritumoral TLOs are positioned just outside the cancer tissue or in the periphery of the cancer (within the cancer-invasive area). Intratumoral TLOs are much rarer than peritumoral TLOs in common types of cancer, but the frequency of intratumoral TLOs varies depending on the tissue of cancer origin and the tumor type. If intratumoral TLOs are relatively abundant in a tumor type that usually shows only a low frequency of them, this suggests that the tumor is a limited or rather specialized case.

### Structures of TLOs

Tertiary lymphoid organs have a structure similar to that of lymph nodes or Peyer’s patches. In addition to the histology, the constituent cells of TLOs and the molecules they express are quite similar to those in SLOs, as would be expected (46–48). T-zone T cells are CD62L^+^ and mainly central memory CD4^+^ T cells or naive T cells that accumulate *via* HEVs from the blood stream. The T cell area also contains immature and CD208^+^ mature DCs. The density of HEVs is strongly correlated with the density of CD3^+^ T cells, CD8^+^ T cells, CD20^+^ B cells, and CD208^+^ mature DCs (49). The B-cell follicle is composed of a mantle of naive B cells, surrounding a germinal center (GC) composed of highly proliferating B cells and a network of CD21^+^ follicular DCs. Development of the GC structure represents an active immune reaction, and the density of GCs in lung and breast cancers has been significantly correlated with patient outcome (48, 50, 51). In addition to lymphoid chemokines (CCL19, CCL21, CXCL13) and adhesion molecules (ICAM-2, ICAM-3, VCAM-1, MAdCAM-1), CCL17, CCL22, and IL16 are found in TLOs (48, 50–52). One interesting feature in lung cancer is that no NKp46^+^ NK cells are detected in TLOs (47), thus allowing them to be distinguished from SLOs. NK cells and DCs are co-localized in lymph nodes, and their interaction enhances NK cell proliferation, IFN-γ secretion, and cytotoxic function, as well as promoting DC maturation (47).

## Clinicopathological Impact of TLOs in Human Cancers

The presence of TLOs in cancer tissues has been reported to be a favorable prognostic indicator (Table 1) (45, 48, 50–68), although some studies have concluded that this is not always the case (55, 60), or may only apply to exceptional cancers such as renal cell carcinoma (61).

**Table 1:** **Summary of TLOs in human cancers**.

Cancer types	Evaluation	Numbers of case	Location of TLOs	Frequency of TLOs (presence)	Prognostication	Significant association			Reference
						Clinicopathological variables	TIL, TAM	Others
Breast cancer	IHC (PNAd^+^ HEV)	146 (Stage I–III)	Mixed (peritumoral and intratumoral)	ND	Favorable (OS, DFS)	No association (tumor size, grade, nodal metastasis, hormone receptor status, adjuvant chemotherapy, CD34^+^ blood vessel within cancer stroma)	CD3^+^T, CD8^+^T, FOXP3^+^ cells, ratio of FOXP3/CD3^b^, CD20^+^B	[Gene expression] related to Th1 cell orientation, cytotoxic granules, lymphoid chemokines, and T cell homing receptors	(48, 52)
	Histology and IHC with gene expression (Tfh)	794 (Stage I–III, ER^−^HER2^−^ 163, HER2^+^ 120, ER^+^HER2^−^ 510)	Mixed (peritumoral and intratumoral)	ND	Favorable (DFS) in all patients, HER2^+^, or ER^+^HER2^−^ patients	ND	Tfh, Th1	[Gene expression] CXCL13	(51)
	Gene expression (Tfh, CXCL13)	996 [preoperative chemotherapy (+)]	Mixed (peritumoral and intratumoral)	ND	Favorable	ND	ND	ND	(51)
	Histology and IHC (CD3^+^T, CD20^+^B)	290 (Stage I–III, invasive carcinoma 257, DCIS 33)	Peritumoral	110 (38.6%)	No prognostic	DCIS grade, ER status^b^, PR status^b^, HER2 status, tumor grade, nodal metastasis, histological TIL	ND	ND	(60)
	Recommended histological criteria (68)	769 (Stage I–III, triple negative breast cancer)	Peritumoral	713 (92.7%: minimal 17.2%, moderate 36.2%, abundant 39.4%)	Favorable (OS^a^, DFS^a^)	Histological TIL	ND	ND	(64)
	Recommended histological criteria (68)	447 (Stage I to III, HER2^+^ invasive carcinoma)	Peritumoral	404 (79%: minimal 37.1%, moderate 31.3%, abundant 10.2%)	No prognostic	TIL, ER ALLred score^b^, HER2 IHC score, HER2 copy number, DCIS percentage, HLA-A percentage, HLA-ABC percentage	ND	ND	(63)
Colorectal cancer	Gene expression (12 chemokines)	20 (Stage 0–IV_A_, 10 the highest and 11 the lowest score tumors selected from 326)	Mixed (peritumoral and intratumoral)	ND	Favorable (OS)	No association (sex, tumor grade, tumor site, location, MSI-H/MSS status, tumor stage)	ND	(Gene expression) related to cytotoxicity and DC	(57)
	Histology and IHC	418 (Stage I–IV)	Peritumoral (extra-tumoral)	411 (98.3%)	Favorable (OS)	TNM stage^b^, preoperative radiotherapy or chemoradiotherapy^b^, deficient mismatch repair enzyme expression	CD3^+^T, CD8^+^T, FOXP3^+^, CD83^+^ (at invasive front and at stromal); CD3^+^T, CD8^+^T (intraepithelial)		(65)
		149 (Stage I–IV)	Peritumoral (extra-tumoral)	147 (98.7%)					(65)
		351 (Stage II 185, stage III 166)	Mixed (peritumoral and intratumoral)	276 (78.6%)	Favorable (DFS) in stage II; no prognostic (DFS) in stage III	PNAd^+^HEV	CD3^+^T		(58)
	IHC (PNAd^+^ HEV)	62 (Duke’s A and C)	Peritumoral (extra-tumoral), intratumoral	Peritumoral TLO: 49 (79%); intratumoral TLO: <8 (12.9%)	No prognostic	More advanced disease, no association (MSI status)		ND	(55)
Lung cancer	IHC (CD208^+^mature DC)	74 (Stage I to II, ADC 46, SCC 28)	Mixed (peritumoral and intratumoral)	ND	Favorable (OS, DSS, DFS)	No association (sex, smoking history, histology, tumor grade, TNM stage, fibrosis, necrosis, Ki-67 tumor cells)	CD3^+^T, ratio of CD4/CD8, T-bet^+^, CD20^+^B	ND	(46, 50, 59)
	IHC (CD208^+^mature DC, follicular CD20^+^B)	122 (Stage III with neoadjuvant chemotherapy)	Mixed (peritumoral and intratumoral)	ND	Favorable (DSS)	ND	ND	An adaptive and specific humoral immune response (+)	(50)
	IHC (CD208^+^mature DC)	458 (Stage I–IV, ADC 241, SCC 111, others 18, ND 6)						(Gene expression) related to cytotoxicity and Th1	(62)
Germ cell tumor	Histology	6 (Intracranial germinaom 2, seminoma 3, dysgerminoma 1)	Intratumoral TLO	ND	ND	ND	ND	an adaptive and specific humoral immune response (+)	(66)
MALT lymphoma	Histology	20 (salivary gland)	ND	ND	ND	ND	ND	ND	(53)
Skin Merkel cell carcinoma	Histology	21 (Stage I to IV)	Mixed (peritumoral and intratumoral)	8 (38%)	Favorable (DFS)^a^, no prognostic (OS)	No association (age, sex, TNM stage, extension status)	Ratio of CD8/CD4 (at tumor periphery)	ND	(54)
Oral squamous cell carcinoma	Histology and IHC (CD3^+^T, CD20^+^B, PNAd^+^HEV)	80 (Stage I to IV)	Peritumoral predominant	17 (21%)	Favorable (DSS)^a^	No association (age, sex, smoking history, alcohol consumption, tumor site, tumor grade, TNM stage, treatment, HPV status)	ND	ND	(67)
Pancreatic cancer	Histology and IHC (CD3^+^T, CD20^+^B, PNAd^+^HEV)	308 (Stage I to IV)	Intratumoral and peritumoral	Intratumoral TLO 49 (12.9%), peritumoral TLO 308 (100%)	Only intratumoral TLO: favorable (OS, DFS)	Only intratumoral TLO: tumor grade^b^, venous invasion^b^	Only intratumoral TLO: CD3^+^T, CD4^+^T, CD8^+^T, ratio of FOXP3/CD4^b^, CD163^+^M2^b^	Only intratumoral TLO: (gene expression) related to Th1 and Th17	(45)
	Histology and IHC (CD3^+^T, CD20^+^B, PNAd^+^HEV)	226 (Stage I to IV)	Intratumoral and peritumoral	Intratumoral TLO 37 (16.4%), peritumoral TLO 308 (100%)	Only intratumoral TLO: favorable (OS, DFS)	ND			(45)
Renal cell carcinoma	IHC (CD208^+^mature DC)	135 (Clear cell RCC), 51 ccRCC lung metastasis	Peritumoral?	ND	No prognostic (TLS-DC, OS, DFS), unfavorable (NTLS-DC, peritumoral, OS, DFS)	TLS-DC: PD-1^+^ cells^b^, PD-L1, and/or PD-L2^+^ tumor cells^b^	ND	ND	(61)
Cutaneous metastasis of malignant melanoma	Histology and IHC (CD3^+^T, CD20^+^B, PNAd^+^HEV)	29	Mixed (peritumoral and intratumoral)	7 (24% complete TLO), 6 (20% incomplete TLO)	ND	ND	ND	ND	(56)

*^a^By only univariate analysis*.

*^b^Negatively associated*.

*ADC, adenocarcinoma; DCIS, ductal carcinoma in situ; DFS, disease-free survival; DSS, disease-specific survival; HPV, human papilloma virus; IHC, immunohistochemistry; MSI-H, microsatellite instability – high; MSS, microsatellite stable; ND, not determined; OS, overall survival; PNAd, peripheral node addressin; SCC, squamous cell carcinoma; TAMs, tumor-associated macrophages; TILs, tumor-infiltrating lymphocytes*.

### Evaluation of TLOs

**Figure 2:**
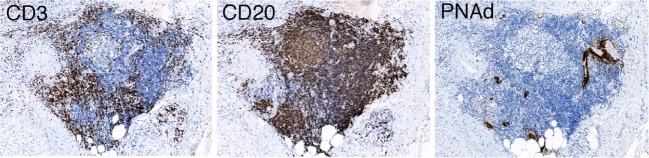
**Immunohistochemistry detecting a TLO having B-cell follicles, T-cell zones, and HEVs detected by immune-labeling for CD20^+^ cells, CD3^+^ cells, and PNAd^+^ vessels, respectively (45)**.

It is important to detect the presence of TLOs in both tumors and the tissues surrounding them. However, there is still no consensus regarding the best method for evaluation of TLOs as different approaches may be needed according to the types of cancer or the tissues from which they develop. Recent studies have detected TLOs using a combination of histological and immunohistochemical methods according to whether the TLOs have B-cell follicles, T-cell zones, and HEVs detected by immune-labeling for CD20^+^ cells, CD3^+^ cells, and PNAd^+^ vessels, respectively (Figure 2). This offers a basic approach that can evaluate the clinicopathological and biological characteristics of TLOs from a neutral viewpoint. Other methods have adopted a morphological approach, or the use of specific markers such as lymphoid aggregates with CD208^+^ mature DCs, or the expression profiles of chemokine genes. These are good biomarkers for detection of TLOs with active immune reactions in lung, breast (48, 59), or colon cancers that are significantly correlated with a better patient outcome (57). One potential problem is that these markers are not always specific, for example the detection of CD208^+^ cells in cancer tissue as representative of the presence of TLOs can be applied to only limited types of cancer where all the CD208^+^ cells are mature DCs located within TLOs. Lung cancer is a good example of this, although in renal cell carcinoma CD208^+^ cells are present in non-TLO stroma (61).

Recently, the International TIL Working Group (2014) has informally recommended a method for evaluating TLOs in breast cancer within the existing method for evaluation of TILs (69). In breast cancer, it has been pointed out that TLOs are typically localized in the area surrounding the tumor, and may be localized in normal tissue directly adjacent to the tumor. However, only a limited population of breast cancers has apparent intratumoral TLOs. TILs are counted within the borders of the invasive tumor and exclude TLOs. Lee et al. have evaluated TLOs using this recommended method and observed that TLOs were present mainly around carcinoma *in situ* (CIS) and in adjacent terminal duct lobular units (63, 64).

### Frequency of TLOs

The frequency of TLOs varies, and tends to be dependent upon where the TLOs are located and the types of cancer (Table 1). At least one peritumoral TLO has been found in more than 90% of cases of colorectal, pancreatic, and breast cancer, while intratumoral TLOs have been observed in only about 15% of colorectal and pancreatic cancers. About 20–40% of skin and oral mucosal cancers have TLOs, where the surfaces of background tissues are covered by squamous cell epithelium. TLOs in oral squamous cell carcinoma are found mainly in the peritumoral stroma within 0.5 mm from the tumor front, in lymphocyte-rich subepithelial areas (67). The density of TLOs in breast cancer shows a marked reduction from DCIS to invasive carcinoma, although other studies have obtained different findings (60).

### Prognostic Impact

The presence of TLOs is usually indicative of a favorable prognosis, although a few reports have suggested it may have a rather negative prognostic impact (Table 1). Bento et al. reported that the presence of extra-tumoral TLOs in colorectal cancer was significantly associated with more advanced disease (55). Figenschau et al. considered that cases of breast cancer associated with TLOs had a higher tumor grade and a high frequency of lymph nodal metastasis (60). These inconsistent results may explain why no universally accepted method for evaluating TLOs has emerged.

The location of TLOs has not been considered in the majority of previous studies. We have evaluated the clinicopathological impact of both peritumoral and intratumoral TLOs in two different cohorts of patients with pancreatic cancer (*n* = 308, 226). Though the presence and density of peritumoral TLOs were not prognostic, the presence of intratumoral TLOs was an independent prognostic factor. In only five cases (1.6%) that showed a higher density of intratumoral TLOs, four of the patients survived without recurrence at least 7 years after the surgery even though one of them had stage 2A disease, three were stage 2B, and one was stage 4 (45).

### Association with Clinicopathologic Variables

The presence of TLOs is basically independent of various clinicopathologic factors in several types of cancer (Table 1). Sometimes a few factors may be significantly associated with TLOs, although no definite tendency has yet emerged. The microsatellite instability (MSI) subset of colorectal cancer has been shown to have an immunogenic character with massive TIL, although the relationship between TLOs and MSI is controversial; in colorectal cancers, two studies found no significant association (55, 57) and one study demonstrated a significant association whereby a more marked Crohn’s-like lymphoid reaction (extra-tumoral TLOs) was significantly associated with deficient expression of the mismatch repair enzyme (65).

### Association with the Immune Microenvironment

The presence and density of TLOs are significantly correlated with immune reaction in many cancers, although there are differences in degree among studies or cancer types (Table 1). TILs (TLOs are usually not counted as TILs) are better indicators of the immune microenvironment, as they lie within the tumor that are detected by histological examination, although it is impossible to avoid contamination from lymphocytes infiltrating into tissues surrounding the cancer invasive area or lymphocytes in intratumoral TLOs in assays such as flow cytometry and RT-PCR using cells or tissues prepared on the basis of macroscopic findings.

Tertiary lymphoid organ presence and density are associated with mainly Th1- and cytotoxicity-related cellular immune reactions that commonly occur in cancers of the breast, colorectum, lung, and pancreas (Table 1). TLO density is also associated with FOXP3^+^ cells but negatively associated with the FOXP3^+^/CD3^+^T cell ratio or the FOXP3/CD4^+^T cell ratio, and also M2 macrophages in breast and/or pancreatic cancer, being consistent with features mentioned above (45, 48). In the tumor microenvironment associated with TLOs, Th1- and cytotoxicity-related genes are commonly expressed, whereas DC-related genes are expressed in colorectal cancer and Th17-related genes in pancreatic cancer. Th2- and immune inhibition-related genes are not significantly associated with TLOs. The presence and density of TLOs are associated with CD20^+^ B cells. In breast and lung cancers, the size and density, or numbers, of B-cell follicles or GCs are significantly correlated with favorable outcome (50, 51). Furthermore, in lung cancer and germ cell tumors, the presence of B-cell follicles in TLOs shows that the machinery for GC somatic hypermutation and class switch recombination is activated, along with the generation of plasma cells (50, 66). It has been thought that B cells play an important role in antitumor immunity, perhaps by capturing and presenting tumor antigens to T cells or by generating tumor antigen-specific antibodies that target tumor antigens to DCs expressing receptors for antibody constant regions. Germain et al. have stated that the presence of CD208^+^ mature DCs with a high density of B-cell follicles is a strong indicator of outcome in patients with lung cancers (50). Thus, it appears that a high density of TLOs is a good biomarker of a tumor immune microenvironment where active cellular and humoral immune reactions are occurring.

A recent study has also shown that Treg cells actively restrain effector T cells within tumor-associated TLOs. Localized Treg cell depletion in a murine model of lung adenocarcinoma triggers robust effector T cell responses and tumor destruction, suggesting that, in this model, Treg cells in TLOs actively restrain anti-tumor immunity (70). Therefore, in order to enhance the effects of immunotherapy, it is recommended that Treg depletion should be performed.

## Formation, Maintenance, and Induction of TLOs in Cancer Tissues

### Formation of SLOs and TLOs

The mechanism of SLO formation has been studied actively, and is considered to share a number of features with TLO formation. Details can be found in another review (29–34, 71). Molecular and cellular mechanisms exist for the development of SLOs (29–34, 71). One pathway mainly for organogenesis of lymph nodes is initiated by interaction between CD3^−^CD4^+^CD45^+^ lymphoid tissue inducer (LTi) cells and stromal organizer (STo) cells at the lymph node anlagen. These specialized cells are of hematopoietic and mesenchymal lineage, respectively. Retinoic acid, probably derived from nerve fibers, induces the expression of CXCL13 in stromal cells. LTi cells accumulate in response to local expression of CXCL13 to form the first cell clusters. In response to IL-7 and TNFSF11, LTi cells are induced to secrete lymphotoxin (LT) α_1_β_2_. Interaction of LTα_1_β_2_ expressed on LTi cells with the LTβ receptor (LTβR) expressed on stromal cells allows the latter to differentiate into STo cells, resulting in secretion of the lymphoid chemokines CCL19, CCL21, and CXCL13 to recruit hematopoietic cells and increase the expression of VCAM-1, ICAM-1, and MAdCAM-1 to ensure lymphocyte retention. Chemokines CCL19 and CCL21 interact with their receptor CCR7 to recruit T cells and DCs, and chemokine CXCL13 interacts with the chemokine receptor CXCR5 to recruit B cells. STo cells also secrete VEGF-C, FGF-2, and HGF, which promote development of the lymphatic vasculature and HEVs. STo cells also differentiate into stromal cell lineages including follicular DCs, fibroblastic reticular cells, and marginal reticular cells, which populate lymph nodes and contribute to SLO function. Another pathway operates mainly for Peyer’s patch formation. CD11c^+^ cells engage RET ligand expressed on gut, and RET-dependent signaling leads to expression of LTα_1_β_2_ by the CD11c^+^ cells. Interaction of these CD11c^+^ cells and LTβR^+^ stromal cells allows the latter to differentiate into STo cells, followed by steps similar to those responsible for formation of lymph nodes. It has been speculated that the mechanism responsible for formation of TLOs is similar to that for SLOs, especially lymph node and mucosal lymphoid tissues, since there are many features of TLOs that are common to the formation of SLOs. Th17, γδT cells expressing IL-17A, or innate lymphoid cell 3 (ILC3) may substitute for LTi cells for development of TLOs (72, 73). These cells share common features with LTi cells, e.g., production of common cytokines such as IL-17A, IL-22, LTβ, TNF, and GM-CSF. T follicular helper (Tfh) cells expressing CXCL13 are also implicated in the regulation of TLOs, representing a key initiator of lymphoid organogenesis that functions upstream of LTβR signaling, promoting B-cell activities, and supporting the generation of high-affinity antibodies at GCs (72, 74, 75). Instead of STo cells, stromal tissue cells such as synovial fibroblasts (e.g., in rheumatoid arthritis) contribute to TLO formation. CXCL13 can be produced by marginal reticular cells, Tfh and follicular DCs, as well as some monocytes/macrophages, a subset of memory T cells, activated B cells, some endothelial cells, stromal cells, or epithelial cells in inflammatory foci. Chemokines, CXCL13, CCL19, CCL21, and CXCL12 are involved in not only the initiation of TLO development, but also maintenance of the highly organized cellular architecture of established SLOs and TLOs.

### Formation and Maintenance of TLOs in Cancer Tissues

How do TLOs develop in human cancer tissues? Is the process different from that occurring in chronically inflamed tissue? Currently there is still no complete answer, although it likely involves (1) the state of antitumor immunity (tumor immunogenicity and immune microenvironment) and (2) the state of tissue structures necessary for formation/maintenance of TLOs.

A number of previous studies have indicated that an active immune response is usually present in background tissues with TLOs, both in cancer and chronic inflammation. The structures and contents of TLOs are comparable between these two situations, although cancer-associated TLOs show a high density of Tregs and absence of NK cells. The location of TLOs is another significant point, as mentioned above. In contrast to the evenly distributed TLOs in chronically inflamed tissues, TLOs in the majority of cancers are present in the area surrounding the invasive lesion, i.e., they are peritumoral TLOs. In non-invasive breast cancer (DCIS) (63, 64), it is interesting that non-invasive cancerous ducts or lobules, including peripheral ducts, are surrounded by TLOs, bearing a histological resemblance to a chronic autoimmune reaction. In invasive cancers, almost the entire cancer tissue is surrounded by TLOs, and intratumoral TLOs are rarely present. Etiologically, TLO formation is expected to occur in tissues with active and continuous immune reactions involving active inflammation. This situation is common in tissues surrounding cancer, since the foreign antigens of cancer cells are presented continuously, tissue destruction caused by cancer invasion becomes a trigger of inflammation, and healthy non-cancerous tissue structures (blood vessels, nerve fibers, extracellular matrix, etc.) remain.

Arterioles and venules, which are small-sized arteries and veins, respectively, are associated with TLOs, and nerve fibers are usually found in TLOs in pancreatic cancers and chronic pancreatitis (45). TLOs, which develop in the pancreatic parenchyma in both chronic pancreatitis and pancreatic cancer, are always found in the interlobular spaces, where arterioles, venules, and relatively large nerve fibers are confined (45). It is speculated that these arterioles, venules, and nerve fibers are necessary for formation and/or maintenance of TLOs. The high frequency of arterial or venous invasion by cancer cells reduces the densities of arteries, arterioles, veins, and venules in the pancreatic cancer stroma. Common pancreatic cancers that have a high frequency of venous or arterial invasion lack intratumoral TLOs regardless of whether peritumoral TLOs are present. Conversely, intratumoral TLOs are found in pancreatic cancers with a low frequency of venous or arterial invasion (45).

**Figure 3:**
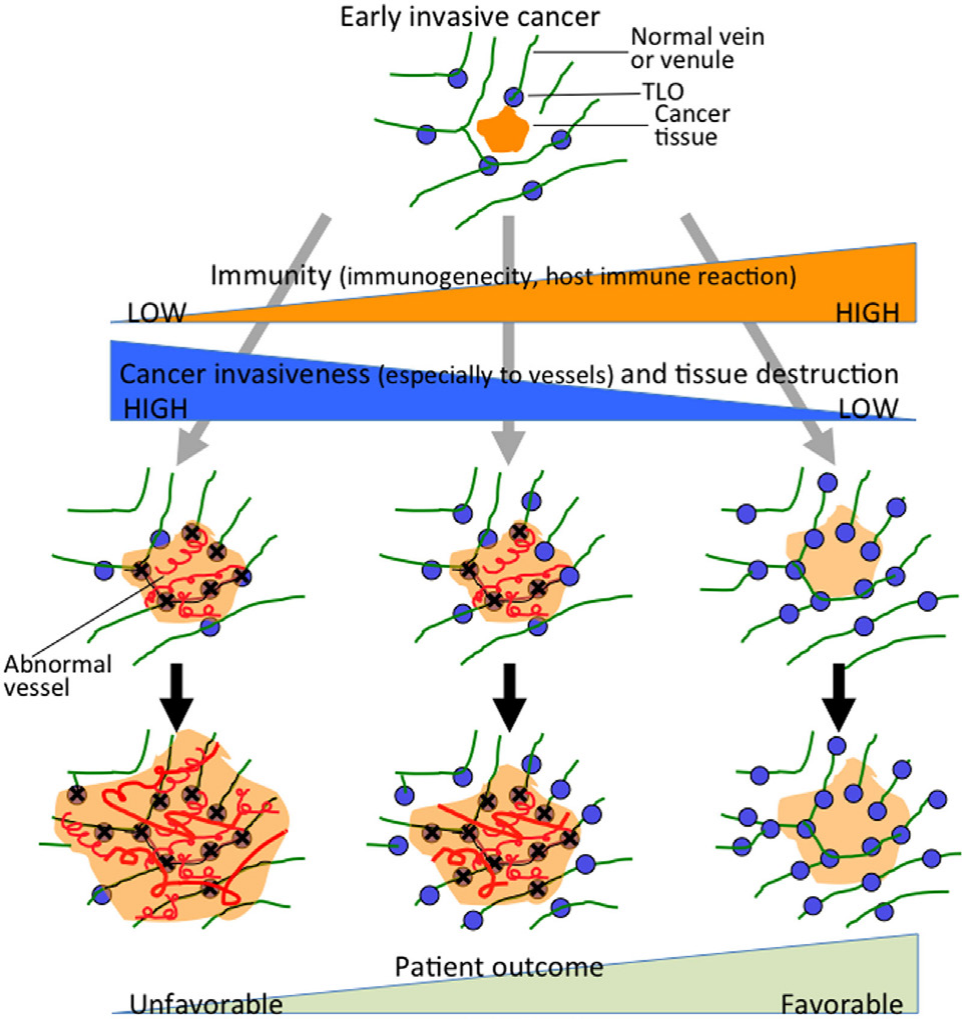
**Hypothesis of TLO formation/maintenance in cancer tissue, which likely involves (1) the state of antitumor immunity (tumor immunogenicity and host immune reaction) and (2) cancer invasiveness especially to vessels and tissue destruction that significantly affects the state of tissue structures necessary for formation/maintenance of TLOs**. TLOs develop in the locations of venules in association with arterioles, venules, and nerve fibers (45). Cancer tissue that has become remodeled, showing absence of functional vascular networks replaced by abnormal blood vessels after invasion of cancer cells (center and left). Peritumoral TLOs can be induced if appropriate immune stimuli are present. Cancer tissue with intratumoral TLOs (right) shows a lower degree of cancer invasiveness, especially to vessels, and an active associated immune reaction. It retains relatively intact vascular networks, transporting immune cells, or other molecules into the cancer tissues, thereby rendering the antitumor immune reaction more effective. The scheme can apply to various solid cancers, although tendency of TLO development may be modified by tissue- and tumor type-specific properties.

A high frequency of venous invasion is found in TLOs at the invasive front. It is speculated that peritumoral TLOs in pancreatic cancer are destroyed and dispersed by cancer invasion, mainly venous invasion, and that other TLOs just outside the invasive front are induced to form. These in turn become surrounded by newly invasive cancer tissues, thus becoming new peritumoral TLOs, which are again subjected to invasion and destruction by further cancer invasion. These processes are repeated, resulting in cancer tissue being surrounded by peritumoral TLOs, whereas intratumoral TLOs are absent (Figure 3). Peritumoral TLOs can be induced if appropriate immune stimuli are present, since arterioles, venules, and nerve fibers remain intact in the surrounding tissue outside the cancer-invasive area, being consistent with the above hypothesis.

It is noteworthy that pancreatic cancers with intratumoral TLOs show peculiar clinicopathological behavior, with a lower degree of cancer invasiveness, especially to venules, and an active associated immune reaction. In addition, the tumor microenvironment has abundant arterioles and venules without cancer invasion. Furthermore, there are relatively many blood vessels (mainly capillaries) that appear to be morphologically and immunohistochemically intact; there is higher expression of VE-cadherin, which is known to be abundant in quiescent and mature vessels (76, 77), and the density of endothelial cells in the abnormal blood vessels (so-called tumor vessels) is lower, lacking a covering of pericytes positive for α-smooth muscle actin (45). It is suggested that at least partly functional vascular networks are retained, transporting immune cells or other molecules into the cancer tissues, thereby rendering the antitumor immune reaction more effective, although vascular density is exceptionally low within the pancreatic cancer tissue in general (78). Murine vascular studies have shown that vascular normalization in tumors enhances the influx of immune effector cells into the tumor parenchyma and markedly prolongs the survival of tumor-bearing mice (79, 80). Pancreatic cancers with intratumoral TLOs might offer a higher chance of effector immune cells, drugs, or effector molecules coming into contact with cancer cells as a result of immunotherapy, chemotherapy, or molecular targeting therapy.

Induction of TLOs after antitumor vaccination has been reported. Two weeks after vaccination with a granulocyte-macrophage colony-stimulating factor (GM-CSF)-secreting pancreatic tumor vaccine (GVAX), intratumoral TLOs with an active cellular and humoral immune response were induced (81). However, it is not clear whether the postvaccination induced TLOs are identical to naturally occurring TLOs, since no comparison of the induced TLOs with naturally occurring TLOs is provided and HEV status is not mentioned in these induced TLOs (81). The induction of TLOs alone did not accurately predict the postvaccination patient outcome, since TLOs were induced in most vaccinated patients’ tumors (85%), although not all tumors from patients with short survival lacked induction of TLOs. Several of the 12 chemokines associated with TLOs in malignant melanoma and colorectal cancer (57, 82) expressed but downregulated in the induced TLOs in patients who have prolonged survival and elevated ratio of effector T cells to Tregs. Meanwhile, TLOs with an active immune reaction were reportedly induced in high-grade cervical intraepithelial neoplasias (CIN2/3) after intramuscular vaccination with HPV16 E6/E7 antigens (83). CIN2/3 can develop cervical squamous cell carcinoma, although no stromal invasion of cancer cells is found. Postvaccination TLOs were induced in the stroma subjacent to residual intraepithelial lesions. The first example is a TLO induced after the pancreatic cancer-associated remodeled tissue, while the second example shows TLOs develop in non-remodeled (but inflammatory) tissue next to the CIN. The second example is similar to the situation at the development of TLOs in persistent active chronic inflammation. It requires further study how TLOs are induced after vaccination more profoundly in the cancer-associated remodeled tissue. Although not all vaccination trials have been able to induce TLOs, these successes might provide clues to the molecular mechanisms occurring in developing TLOs, in addition to development of therapeutic interventions.

### HEV Formation and Maintenance

High endothelial venules are specially differentiated vessels that play important roles in the formation of lymphoid organs through accumulation of naive and central memory lymphocytes or other immune cells including DCs by providing an apparatus for extravasation of these cells from the blood stream into lymphoid organs (35–38). For this activity, HEVs specifically express and produce sulfated carbohydrate ligands, l-selectin ligands, and some adhesion molecules such as ICAM-1, VCAM-1, or MAdCAM-1 (36–44). HEV cells do not express lymphoid chemokines (CCL19, CCL21, and CXCL13) but present them at luminal surfaces through binding to scaffold molecules. Thus, HEVs are necessary for active and functional lymphoid organs.

Several studies have revealed that continuous engagement of LTβR on HEVs by LTα1β2^+^ cells is critical for the induction and maintenance of HEV gene expression and HEV cell morphology (84, 85). In mice, the major sources of LTα1β2 for HEV regulation in lymphoid tissues are CD11c^+^ DCs and B cells (86–88). CD11c^+^ cells and activated B cells contribute to an increase of VEGF production, resulting in proliferation of endothelial cells in lymph nodes. Retention of routes for the recruitment of CD11c^+^ DCs into TLOs might be necessary in order to maintain the HEV phenotype in TLOs. It is assumed that if these routes are shut down by cancer invasion, HEV function will fail and the cellular content will be reduced, eventually leading to a decline in TLOs. Intratumoral HEVs would be expected to fail easily. In fact, in colorectal cancer, HEVs composed of flattened, atypical endothelial cells without lymphoid aggregates are observed within the tumor, whereas HEVs composed of normal tall columnar to cuboidal-shaped endothelial cells with lymphoid aggregates are found in peritumoral areas (55).

## Concluding Remarks

Reports that have accumulated so far suggest that tumor-associated TLOs in many types of cancers play roles in the initiation and maintenance of active cellular and humoral immune responses against the cancers. Indeed, the presence of TLOs is significantly correlated with a favorable patient outcome and with a tumor immune microenvironment showing responses involving cellular and humoral immunity. The location of TLOs varies according to the type of cancer, and is important for evaluating the pathological significance of TLOs, i.e., whether they develop as a response to the cancer or as a result of secondary inflammatory changes caused by cancer invasion. Although the developmental mechanisms of TLOs are thought to be shared with those of SLO formation, we need to understand them in more detail, particularly the differences between TLOs that develop in non-cancerous tissues and those that develop in tissues remodeled by cancer invasion, and also the factors that trigger TLO development. In order to achieve this, we have to understand the real tumor-associated TLOs more and more with considering relationships of TLOs with cancer cells, and other stromal components including blood vessels, fibroblasts, and extracellular matrix. Unfortunately, any correlation between TLO formation and clinical outcome does not provide much information about the mechanism involved, and therefore both observational studies of human cancer and functional studies using reliable models will be required. It is anticipated that the presence (or higher density) of TLOs may be applicable as not only a prognostic marker but also a biomarker for selection of patients suitable for immunotherapy and/or for monitoring of patients during therapeutic intervention.

## Author Contributions

Conception and design of the work: NH. Acquisition of data: NH, YI, and RY-I. Writing, review, and/or revision of the manuscript: NH, YI, and RY-I. Final approval of the version: NH, YI, and RY-I. Administrative, technical, or material support: NH, YI, and RY-I. Study supervision: NH.

## Conflict of Interest Statement

The authors declare that the research was conducted in the absence of any commercial or financial relationships that could be construed as a potential conflict of interest.
